# Minimal change disease after multiple wasp stings 

**DOI:** 10.5414/CNCS110369

**Published:** 2022-01-24

**Authors:** Yasmine S. Humeda, William L. Clapp, Humam Humeda

**Affiliations:** 1 Department of Medicine, Tallahassee,; 2 Department of Pathology, Immunology, and Laboratory Medicine, University of Florida College of Medicine, Gainesville, and; 3 Renalus Center for Kidney Care, Pensacola, FL, USA

**Keywords:** minimal change disease, wasp, nephrotic syndrome, acute renal failure

## Abstract

Acute renal failure is a well-known but uncommon complication of wasp stings. In rare instances, nephrotic syndrome (NS) has also been reported in association with wasp envenomation. The occurrence of minimal change disease (MCD) as a consequence of wasp stings is even less common, with only 1 case reported to date. We report a case of a 67-year-old man, with previously normal kidney function, presenting with acute renal failure with underlying NS due to biopsy-proven MCD, 1 month following numerous wasp stings. Despite treatment with corticosteroids, the patient required hemodialysis and treatment with loop diuretics and prednisone for 6 months until complete resolution. The patient remains free of NS, with normal renal function 3 years following remission.

## Introduction 

Allergic reactions, including anaphylaxis, to bee and wasp stings have been documented since ancient times and include varying levels of severity, from localized cutaneous symptoms to systemic reactions [1]. Insect venoms of the order Hymenoptera – including bees (honeybees), vespids (yellowjackets, wasps), and stinging ants (fire ants) – can also result in direct toxic injury to tissues [2, 3]. Renal injury is a known consequence of bee and wasp stings [4]. Toxic envenomation by honeybees usually requires hundreds to thousands of stings to cause a clinically important injury, whereas wasp envenomation can be clinically significant with only 20 – 100 stings and can cause renal failure, multiple organ failures, and death [2, 3]. Acute kidney injury (AKI) resulting from complications from wasp stings can yield a mortality rate of 25% within the first few days following envenomation; thus, renal function should be closely monitored during this time period [5]. Under these circumstances, renal damage is usually attributed to acute tubular injury from direct toxicity of the venom or, in the setting of pigment nephropathy, results from hemolysis or rhabdomyolysis and/or acute tubulointerstitial nephritis caused by a hypersensitivity reaction to the wasp venom [5, 6, 7, 8]. In rare instances, nephrotic syndrome (NS) has also been reported in association with wasp envenomation [9, 10]. The occurrence of minimal change disease (MCD) as a consequence of wasp stings is even less common. To our knowledge, only 1 case has been reported to date [9]. We report a case of a 67-year-old man presenting with acute renal failure with underlying NS due to biopsy-proven MCD, 1 month following numerous wasp stings. 

## Case report 

**Figure 1:**
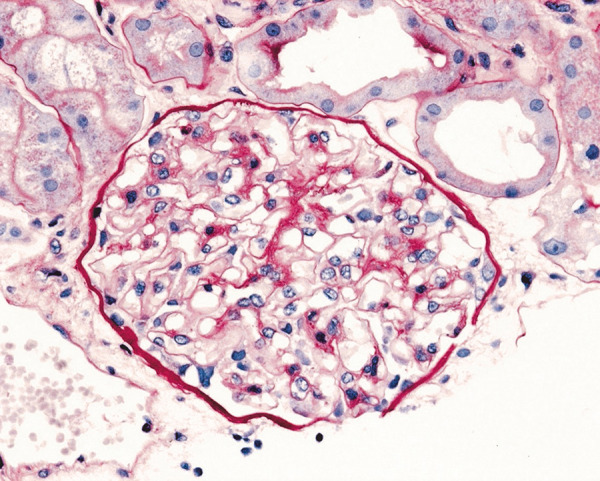
Light microscopy of renal biopsy specimen depicting normocellular glomerulus with patent capillaries in a patient presenting with acute renal failure and nephrotic-range proteinuria (periodic acid-Schiff stain, original magnification × 400).

**Figure 2:**
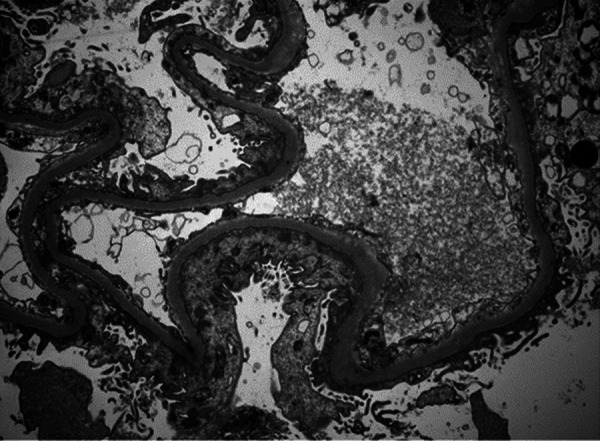
Electron microscopy revealing diffuse podocyte foot process effacement and microvillous transformation. No electron-dense deposits are present (original magnification × 8,000).

A 67-year-old man presented to the emergency department with a subacute onset of diffuse upper and lower extremity swelling and decreased urinary output, 1 month following nearly 100 wasp stings. The patient reported that he had presented to an urgent care facility for localized swelling at the sting sites immediately following the attack, and a 1-week course of corticosteroids was initiated to improve his swelling. However, during the following weeks, he noted a progressive increase in pain and swelling in both of his arms and legs following the completion of treatment, prompting his visit to the emergency department. His past medical history was notable for hypertension and hypothyroidism for which he was taking metoprolol 50 mg and levothyroxine 50 µg, respectively. He had no prior history of kidney disease or allergies. The patient denied use of non-steroidal anti-inflammatory drugs (NSAIDs), D-penicillamine, mercury, gold, lithium, or tyrosine-kinase inhibitors (TKIs). He also denied exposure to toxins or recent immunizations. The patient was up-to-date with all age-appropriate recommended malignancy screenings. Physical examination revealed prominent edema involving bilateral upper and lower extremities and an elevated blood pressure of 172/72 mmHg. Two months prior, the patient’s baseline serum creatinine levels were normal (< 1 mg/dL). At the time of evaluation, he was found to have AKI with a serum creatinine level of 3.5 mg/dL. Additional notable laboratory results included a glomerular filtration rate of 16 mL/min, hypoalbuminemia (1.6 g/dL), hypercholesterolemia (260 mg/dL), and hypertriglyceridemia (184 mg/dL). A 24-hour urinary protein collection contained 10,700 mg of protein. White blood cell count was slightly elevated at 13,100/mm^3^ with 94.2% neutrophils, 3.4% lymphocytes, 2.0% monocytes, and 0.0% eosinophils. Urinalysis revealed turbid, orange-colored urine with 3+ blood, 3+ protein, specific gravity of 1.025, 2+ leukocyte esterase, and negative nitrite. Based on the workup, a diagnosis of NS resulting in AKI was made. The patient was started on intravenous methylprednisolone 500 mg daily for 3 days, followed by oral prednisone 60 mg daily, and intravenous furosemide 80 mg daily. On the sixth day of hospitalization, an ultrasound-guided renal biopsy revealed findings characteristic of MCD. Histology revealed a preserved renal cortex and 17 normal appearing glomeruli on light microscopy (Figure 1); absence of glomerular immunoglobulin and complement component staining on immunofluorescence, and diffuse, podocyte foot process effacement and microvillous transformation on electron microscopy with no electron-dense deposits identified (Figure 2). Mild acute tubular injury was characterized by focal tubular dilation, attenuation of the epithelial cells, and proximal tubule periodic acid-Schiff-positive brush border. Potential underlying causes of MCD were investigated. Serology was negative for HIV, hepatitis B and C, syphilis, anti-nuclear antibodies, anti-double-stranded DNA antibodies, anti-myeloperoxidase, anti-proteinase-3, and anti-glomerular basement membrane antibodies, ruling out several infectious and autoimmune etiologies. Chest X-ray and computed tomography scans of the abdomen were normal. A repeat complete blood count was within normal limits. A diagnosis of MCD secondary to wasp envenomation was made, rendered on the renal biopsy findings, clinical and laboratory features, and exclusion of other causes of MCD. 

Despite 13 days of treatment, the patient showed no clinical response. The patient’s edema worsened, which led to bilateral pleural effusions causing significant shortness of breath and requiring dialysis to be administered for 3 weeks. At the time of discharge (30 days after admission), the patient was excreting 7,290 mg/day of protein. Serum creatinine levels decreased to 2.2 mg/dL, blood urea nitrogen was 17 mg/dL, albumin was 1.9 gm/dL, and slight edema was present. Following discharge, dialysis was discontinued, as the patient responded adequately. He was then treated as an outpatient with furosemide 80 mg twice daily and 60 mg prednisone daily, both of which were gradually tapered over 6 months. At that time, the patient had complete resolution of NS, and urine protein levels had decreased to 120 mg/day. Renal function improved to baseline levels: serum creatinine 0.9 mg/dL, albumin 3.4 g/dL, cholesterol 175 mg/dL, and triglyceride 161 mg/dL. Approximately 3 years following remission, the patient remains free of NS with normal renal function. 

## Discussion 

The rare association between Hymenoptera stings (wasp or bee) and NS was first reported by Rytand in 1955 [11]. Additional cases have been described since then; however, most are attributed to bee rather than wasp stings [4, 9, 10, 11, 12, 13, 14, 15]. The occurrence of MCD as a consequence of wasp envenomation is even rarer [9]. 

Honeybee stings are unique in that they are barbed in shape and remain within the victim’s skin, unlike the stings of wasps. The presence or absence of a detached sting is one way to differentiate between the two insects. This can be significant in allergic individuals, as the allergens in each venom type differ between the two insect groups. Bee venom contains phospholipase A2 – a major allergen – as well as peptide melittin and hyaluronidase, whereas wasp venom contains antigen 5, different phospholipases, and hyaluronidase [16]. 

MCD is a common cause of idiopathic NS, accounting for ~ 15% of cases in adults [17]. It is characterized by proteinuria, hypoalbuminemia, generalized edema, and commonly hyperlipidemia. Its name originates from its relatively normal appearance under light microscopy, while the characteristic effacement of podocyte foot processes of MCD are visualized under electron microscopy. Immunofluorescence is typically negative. Numerous etiologic processes have been linked to MCD, such as toxins, immunizations, and allergens. Malignancies that have been implicated include hematologic malignancies and colorectal carcinoma. NSAIDs, D-penicillamine, mercury, gold, lithium, and TKIs are notable drugs associated with MCD. Infectious etiologies include HIV, hepatitis B and C, and syphilis. Autoimmune causes that have been implicated include lupus and diabetes mellitus. However, most cases are idiopathic, arising mostly in otherwise healthy individuals [17]. Although the exact pathogenesis of MCD remains unknown, it has been suggested that an abnormal immune-mediated response plays a significant role [17]. T-cell activation, and resultant cytokine release by immunological stimuli, has been proposed to cause podocyte injury leading to increased glomerular permeability and proteinuria. The toxic venom of insect stings can induce a systemic allergic response leading to immune dysfunction. A notable observation is the association between MCD and atopy, as T-cell proliferation is also responsible for driving allergies [17]. Patients with a history of atopy have a higher susceptibility to MCD; however, our patient did not demonstrate this association, as he had no history of allergies. 

Although indications for renal biopsy in children are limited, early kidney biopsy in adults with NS is essential for determining the therapeutic approach, as MCD is not the most common cause of NS in adults. More common causes of NS following insect stings include mesangial proliferative glomerulonephritis, membranous glomerulonephritis, and focal segmental glomerulosclerosis, all of which carry a different prognosis and treatment than does MCD [17, 18, 19]. The mainstay of therapy for MCD is prednisone due to its immunosuppressive effects [17]. In steroid non-responsive patients, second-line alternatives for immunosuppression include cyclophosphamide, rituximab, calcineurin inhibitors, and anti-proliferatives [17]. Acute management is centered on improving edema through salt and fluid intake restriction and the use of diuretics [13]. Although the majority of cases resolve promptly with steroid treatment, severe cases may require temporary hemodialysis, as in our patient [18]. However, in accordance with the literature, which suggests that acute renal failure from MCD is usually reversible, our patient achieved normal renal function over time [18]. 

AKI following wasp stings is often attributed to toxic-ischemic acute tubular necrosis and acute tubulointerstitial nephritis rather than NS [6, 7, 19]. Although much less common, NS can also result in acute renal injury, especially in the setting of MCD [17, 18]. This is more frequently seen in adults and is attributed to intravascular volume depletion and oliguria [17]. The average length of time between onset of NS and acute renal failure is 4 weeks [20]. Renal biopsy of MCD at the time of acute renal injury shows features of acute tubular injury with loss or thinning of the proximal tubule brush border, interstitial inflammation, and edema, which are consistent with the findings in our patient [18]. 

This case describes a man with previously normal renal function who presented with diffuse edema, AKI, and biopsy-proven MCD following multiple wasp stings. Despite aggressive corticosteroid therapy, his condition continued to decline, and he ultimately required hemodialysis. This represents a unique clinicopathologic presentation of NS due to MCD secondary to numerous wasp stings. Compared with previously reported cases of MCD due to insect stings that presented with the proteinuria and edema of NS, the inciting source of MCD in this case was wasp rather than bee stings. Additionally, the fact that the patient presented with acute renal failure is of clinical significance. It is important for clinicians to be cognizant of this relationship when treating patients with wasp-sting reactions to ensure close monitoring of renal function and to avoid decompensation into severe acute renal failure, as in our case. This case also highlights the importance of early renal biopsy and steroid therapy in the management of these patients. 

## Funding 

There was no support/funding for this report. 

## Conflict of interest 

The authors have no conflict of interest to declare. 

## References

[1] Abrams EM Golden DBK Approach to patients with stinging insect allergy. Med Clin North Am. 2020; 104: 129–143. 31757231 10.1016/j.mcna.2019.08.006

[2] Vetter RS Visscher PK Camazine S Mass envenomations by honey bees and wasps. West J Med. 1999; 170: 223–227. 10344177 PMC1305553

[3] Schmidt JO Clinical consequences of toxic envenomations by Hymenoptera. Toxicon. 2018; 150: 96–104. 29782951 10.1016/j.toxicon.2018.05.013

[4] Reisman RE Unusual reactions to insect stings. Curr Opin Allergy Clin Immunol. 2005; 5: 355–358. 15985819 10.1097/01.all.0000173782.35283.b6

[5] Ambarsari CG Sindih RM Saraswati M Trihono PP Delayed admission and management of pediatric acute kidney injury and multiple organ dysfunction syndrome in children with multiple wasp stings: A case series. Case Rep Nephrol Dial. 2019; 9: 137–148. 31828077 10.1159/000504043 PMC6902257

[6] Zhang R Meleg-Smith S Batuman V Acute tubulointerstitial nephritis after wasp stings. Am J Kidney Dis. 2001; 38:E33. 11728993 10.1053/ajkd.2001.29289

[7] Chao YW Yang AH Ng YY Yang WC Acute interstitial nephritis and pigmented tubulopathy in a patient after wasp stings. Am J Kidney Dis. 2004; 43: e15–e19. 14750120 10.1053/j.ajkd.2003.10.025

[8] Dhanapriya J Dineshkumar T Sakthirajan R Shankar P Gopalakrishnan N Balasubramaniyan T Wasp sting-induced acute kidney injury. Clin Kidney J. 2016; 9: 201–204. 26985369 10.1093/ckj/sfw004 PMC4792632

[9] Zaman F Saccaro S Latif S Atray N Abreo K Minimal change glomerulonephritis following a wasp sting. Am J Nephrol. 2001; 21: 486–489. 11799266 10.1159/000046653

[10] Tauk B Hachem H Bastani B Nephrotic syndrome with mesangial proliferative glomerulonephritis induced by multiple wasp stings. Am J Nephrol. 1999; 19: 70–72. 10085454 10.1159/000013429

[11] Rytand DA Onset of the nephrotic syndrome during a reaction to bee sting. Stanford Med Bull. 1955; 13: 224–233. 14386174

[12] Venters HD Venier RL Worthen HG Good R A. Bee sting nephrosis: A study of the immunopathologic mechanisms. Am J Dis Child. 1961; 102: 688–689.

[13] Olivero JJ Ayus JC Eknoyan G Nephrotic syndrome developing after bee stings. South Med J. 1981; 74: 82–83. 7455752 10.1097/00007611-198101000-00030

[14] Tasic V Nephrotic syndrome in a child after a bee sting. Pediatr Nephrol. 2000; 15: 245–247. 11149119 10.1007/s004670000452

[15] Cuoghi D Venturi P Cheli E Bee sting and relapse of nephrotic syndrome. Child Nephrol Urol. 1988-1989; 9: 82–83. 3251627

[16] Hoffman DR Hymenoptera venom allergens. Clin Rev Allergy Immunol. 2006; 30: 109–128. 16645223 10.1385/criai:30:2:109

[17] Vivarelli M Massella L Ruggiero B Emma F Minimal change disease. Clin J Am Soc Nephrol. 2017; 12: 332–345. 27940460 10.2215/CJN.05000516 PMC5293332

[18] Waldman M Crew RJ Valeri A Busch J Stokes B Markowitz G D’Agati V Appel G Adult minimal-change disease: clinical characteristics, treatment, and outcomes. Clin J Am Soc Nephrol. 2007; 2: 445–453. 17699450 10.2215/CJN.03531006

[19] Kaarthigeyan K Sivanandam S Jothilakshmi K Matthai J Nephrotic syndrome following a single bee sting in a child. Indian J Nephrol. 2012; 22: 57–58. 22279346 10.4103/0971-4065.83742 PMC3263067

[20] Koomans HA Pathophysiology of acute renal failure in idiopatic nephrotic syndrome. Nephrol Dial Transplant. 2001; 16: 221–224. 11158391 10.1093/ndt/16.2.221

